# Cathelicidin protects mice from Rhabdomyolysis-induced Acute Kidney Injury

**DOI:** 10.7150/ijms.52397

**Published:** 2021-01-01

**Authors:** Beatriz Helena Cermaria Soares da Silva, Suely Kubo Ariga, Hermes Vieira Barbeiro, Rildo Aparecido Volpini, Denise Frediani Barbeiro, Antonio Carlos Seguro, Fabiano Pinheiro da Silva

**Affiliations:** 1 Departamento de Emergências Clínicas, Universidade de São Paulo, São Paulo, Brazil; 2 Laboratório de Investigação Médica 12 (LIM12), Hospital das Clínicas HCFMUSP, Faculdade de Medicina, Universidade de São Paulo, São Paulo, Brazil

**Keywords:** antimicrobial peptide, cathelicidin, innate immunity, acute kidney injury, sepsis, rhabdomyolysis, inflammation

## Abstract

**Background:** Cathelicidins are ancient and well-conserved antimicrobial peptides (AMPs) with intriguing immunomodulatory properties in both infectious and non-infectious inflammatory diseases. In addition to direct antimicrobial activity, cathelicidins also participate in several signaling pathways inducing both pro-inflammatory and anti-inflammatory effects. Acute kidney injury (AKI) is common in critically ill patients and is associated with high mortality and morbidity. Rhabdomyolysis is a major trigger of AKI.

**Objectives:** Here, we investigated the role of cathelicidins in non-infectious Acute kidney Injury (AKI).

**Method:** Using an experimental model of rhabdomyolysis, we induced AKI in wild-type and cathelicidin-related AMP knockout (CRAMP^-/-^) mice. Results: We previously demonstrated that CRAMP^-/-^ mice, as opposed wild-type mice, are protected from AKI during sepsis induced by cecal ligation and puncture. Conversely, in the current study, we show that CRAMP^-/-^ mice are more susceptible to the rhabdomyolysis model of AKI. A more in-depth investigation of wild-type and CRAMP^-/-^ mice revealed important differences in the levels of several inflammatory mediators.

**Conclusion:** Cathelicidins can induce a varied and even opposing repertoire of immune-inflammatory responses depending on the subjacent disease and the cellular context.

## Introduction

Acute kidney injury (AKI) is a multifactorial process associated with a substantial increase in the mortality of critically ill patients. The most common risk factors include advanced age, diabetes, chronic kidney disease, volume depletion, sepsis, rhabdomyolysis and exposure to nephrotoxic drugs 1. To date, no drug has been approved for the prevention of AKI, and treatment relies on hemodynamic optimization and dialysis, if necessary 2. Most successful animal studies have tested prophylactic drug regimens; however, AKI is not routinely identified until one or more days post-insult 1, 3. A better understanding of the molecular mechanisms that trigger and perpetuate AKI is crucial for the development of novel biomarkers and therapies.

Glomerular and peritubular endothelial dysfunction, downregulation of tubular reabsorption, cell cycle arrest and cell death are among the mechanisms that have been implicated in the pathophysiology of septic AKI 4, 5. Renal lesions are initiated by pathogen-associated and damage-associated molecular patterns that activate not only immune cells but many other cell types, such as endothelial, glomerular and tubular cells 4, 6, 7. In response to stress, some tubular cells enter cell cycle arrest, a probable protective mechanism, while sodium pumps are relocated, thus suppressing their activity and causing a significant reduction in oxygen consumption 4, 8. In extreme conditions, cells progressively die, a process that further amplifies the systemic inflammatory response. Glomerular function simultaneously declines because of contraction of the mesangial cells, imbalance in the vasomotor tone and sodium-induced activation of detectors. These events lead to the contraction of the afferent arterioles. Meanwhile, the release of inflammatory mediators leads to the redistribution of renal microcirculation, which results in insufficient blood flow to important areas of the kidney 4.

In the case of rhabdomyolysis, the release of contents from damaged skeletal muscle can lead to AKI via 1) hypoperfusion secondary to fluid sequestration in damage tissues; 2) vasoconstriction due to renin angiotensin aldosterone system activation; and 3) renal accumulation of myoglobin and heme derivates, which can result in intratubular obstruction, renal vasoconstriction, inflammation and tubular damage in response to oxidative stress 9, 10. Lipid peroxidation induces mitochondrial damage, with amplification of reactive oxygen species (ROS) levels and cytochrome c release, culminating in caspase 1 and 3 activation and tubular cell apoptosis 10.

Antimicrobial peptides (AMPs) are important factors that balance the immune response by protecting commensal bacteria from invading pathogens, maintaining the sterility of certain tissues and participating in several immunomodulatory processes 11. Cathelicidins and defensins are the most investigated families of AMPs in mammals and are expressed in immune and epithelial cells 11. LL-37 and cathelicidin-related AMP (CRAMP) are the sole cathelicidins in humans and rodents, respectively. Together with other AMPs, such as β-defensin 1, β-defensin 3, lactoferrin, hepcidin and ribonuclease 7, cathelicidins are produced by the renal epithelium and inflammatory cells.

Overall, these AMPs constitute a crucial component in the maintenance of renal homeostasis and avoidance of disease 12, 13. Indeed, CRAMP^-/-^ mice are more susceptible to urinary tract infections 14 and to experimental hemolytic uremic syndrome 15. However, using sepsis (cecal ligation and puncture; CLP), a well-established model of systemic infection, we have demonstrated that CRAMP^-/-^ mice are more resistant to septic shock, providing evidence for the complex mechanisms involved in AMP biology 16.

Here, we induced AKI in wild-type and CRAMP^-/-^ mice, using a model of rhabdomyolysis. The rhabdomyolysis model was induced by a single intramuscular glycerol injection. After induction of AKI, we investigated the renal function and systemic parameters of the wild-type and CRAMP^-/-^ animals to characterize the role of CRAMP and other AMPs in each scenario.

## Materials and Methods

### Mice

Male CRAMP^-/-^ mice with a C57BL/6 genetic background and matched wild-type controls were purchased from The Jackson Laboratory (ME, USA). All experiments were performed with 8-10-week-old animals.

### Rhabdomyolysis model

We induced rhabdomyolysis in mice by injecting 50% glycerol (10 ml/kg of body weight; diluted in saline 0.9%) intramuscularly in the right hind limb. Mice were subjected to 12 hours of water deprivation before the procedure 17-19. Animals were sacrificed 24 hours after the glycerol injection, and plasma and tissue samples were collected for further analyses.

### Renal function and muscle damage parameters

Urea, creatinine, creatine phosphokinase (CPK), calcium, phosphorus, potassium and uric acid plasma levels were measured using colorimetric, UV kinetic or ion selective assays (Roche, Indianapolis, USA).

### Cytokine levels in plasma and kidneys

TNFα, IL-1β, IL-6 and IL-10 plasma levels were measured using the magnetic bead immunoassay Milliplex® and the MAGPIX® System (Merck Millipore, USA). The same cytokines were measured in kidney samples using ELISA kits (R&D systems, USA).

### Antimicrobial peptides (AMPs)

β-defensins 1 and 3 were measured in lung and kidney tissues, α-defensin 5 was measured in ileum tissue, and neutrophil gelatinase-associated lipocalin (NGAL) was measured in kidney tissue using ELISA (R&D systems, USA).

### Light microscopy

Four-mm histological sections of renal tissue were stained with hematoxylin-eosin and examined under light microscope. For the evaluation of renal damage, 20-30 grid fields (x400 magnification) measuring 0.245 mm^2^ were evaluated by graded scores, according to the following criteria: (0), less than 5% of the field showing tubular epithelial swelling, vacuolar degeneration, inflammatory infiltrate, necrosis and desquamation; (1), 5-25% of the field presenting renal lesions; (2), involvement of 25-50% with renal damage; (3), 50-75% of damaged area; (4), more than 75% of the grid field presenting renal lesions (n = 8 mice per group) 20.

### Statistical analysis

Results were analyzed using the Kruskal-Wallis test followed by the Mann-Whitney U test. Results are shown in boxplots. The mortality curve was produced using the Kaplan-Meier method and log rank test. All analyses were performed using R statistical software (www.r-project.org). A p-value < 0.05 was considered significant.

## Results

### CRAMP^-/-^ mice are more susceptible than wild-type mice to rhabdomyolysis

**Figure 1:**
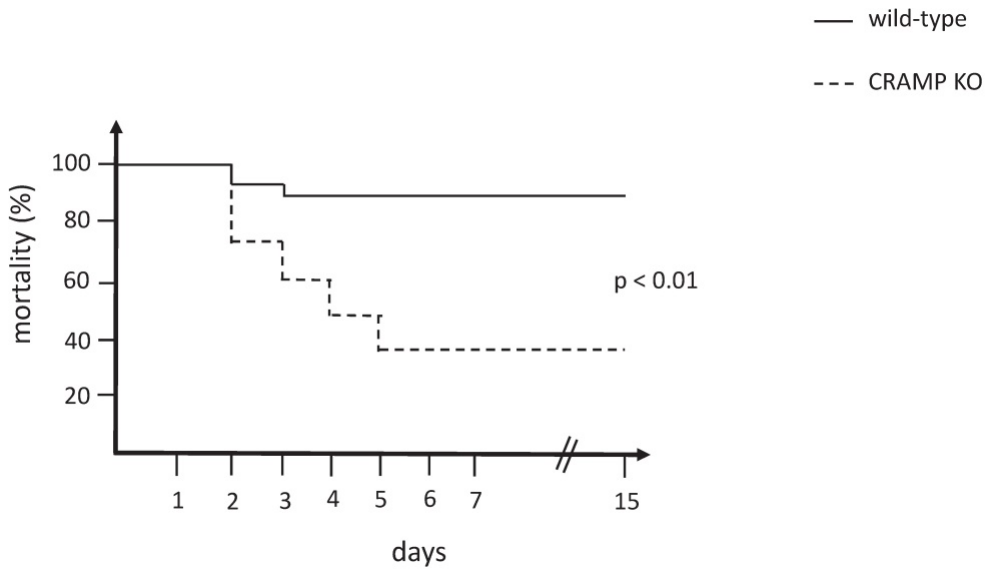
Mortality curve comparing wild-type and CRAMP^-/-^ mice subjected to the rhabdomyolysis model (n = 15 mice per group).

The mortality curve of CRAMP^-/-^ mice was significantly different from the wild-type matched controls (p < 0.01), which revealed that the CRAMP^-/-^ mice died more rapidly when subjected to the rhabdomyolysis model of AKI (**Figure 1**).

### Parameters for renal function impairment and muscle damage are similar in wild-type and CRAMP^-/-^ mice after induction of rhabdomyolysis

**Figure 2:**
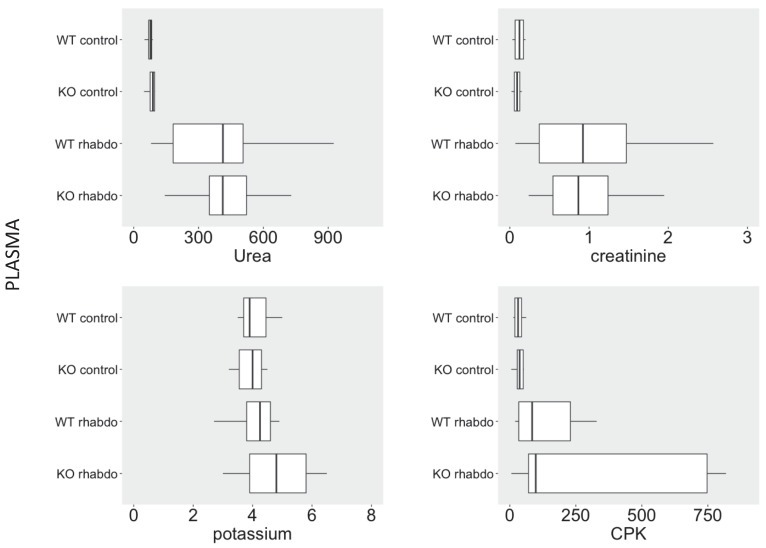
Plasma levels of urea (mg/dL), creatinine (mg/dL), CPK (U/L), potassium (mEq/L), calcium (mg/dL) and phosphorus (mg/dL) in wild-type and CRAMP^-/-^ mice (n = 7-11 animals per group).

Rhabdomyolysis was successful in producing a rapid impairment of renal function in wild-type and CRAMP^-/-^ animals, but similar levels of urea, creatinine, CPK and potassium were found in plasma from wild-type and CRAMP -/- animals submitted to the rhabdomyolysis model (**Figure 2**). Phosphorus, calcium and uric acid levels were also similar in the study groups (data not shown).

### Wild-type and CRAMP^-/-^ mice exhibited similar plasma cytokine levels after induction of rhabdomyolysis

**Figure 3:**
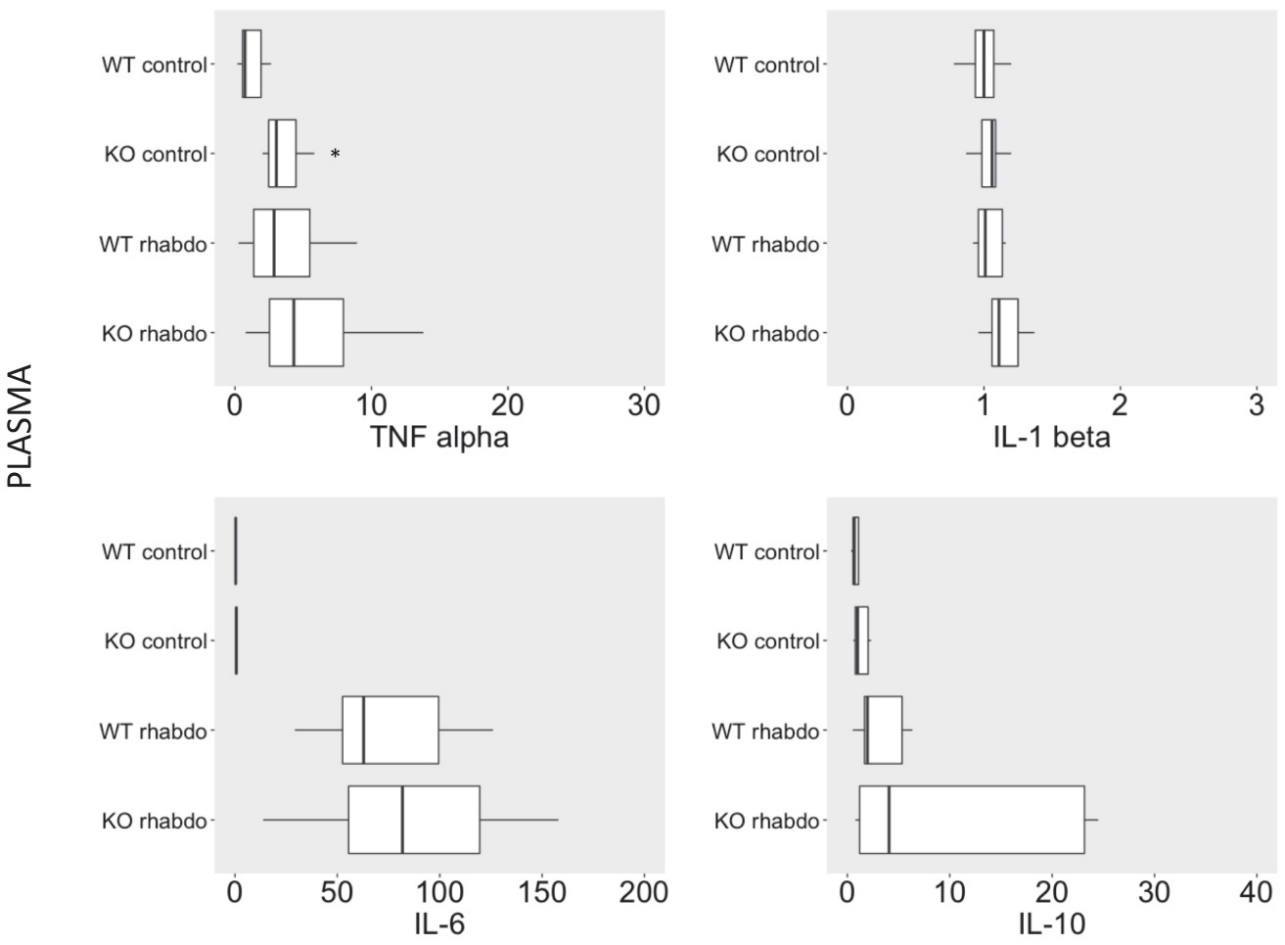
Levels of TNFα (pg/ml), IL-1β (pg/ml), IL-6 (pg/ml) and IL-10 (pg/ml) in plasma from wild-type and CRAMP^-/-^ mice (n = 8-12 animals per group).

No differences were detected between levels of TNFα, IL-1β, IL-6, and IL-10 in plasma from wild-type and CRAMP^-/-^ mice when subjected to the rhabdomyolysis model (**Figure 3**).

### CRAMP^-/-^ mice exhibited significantly higher TNFα, IL-1 β levels and IL-6 in kidney tissue after induction by the rhabdomyolysis model

**Figure 4:**
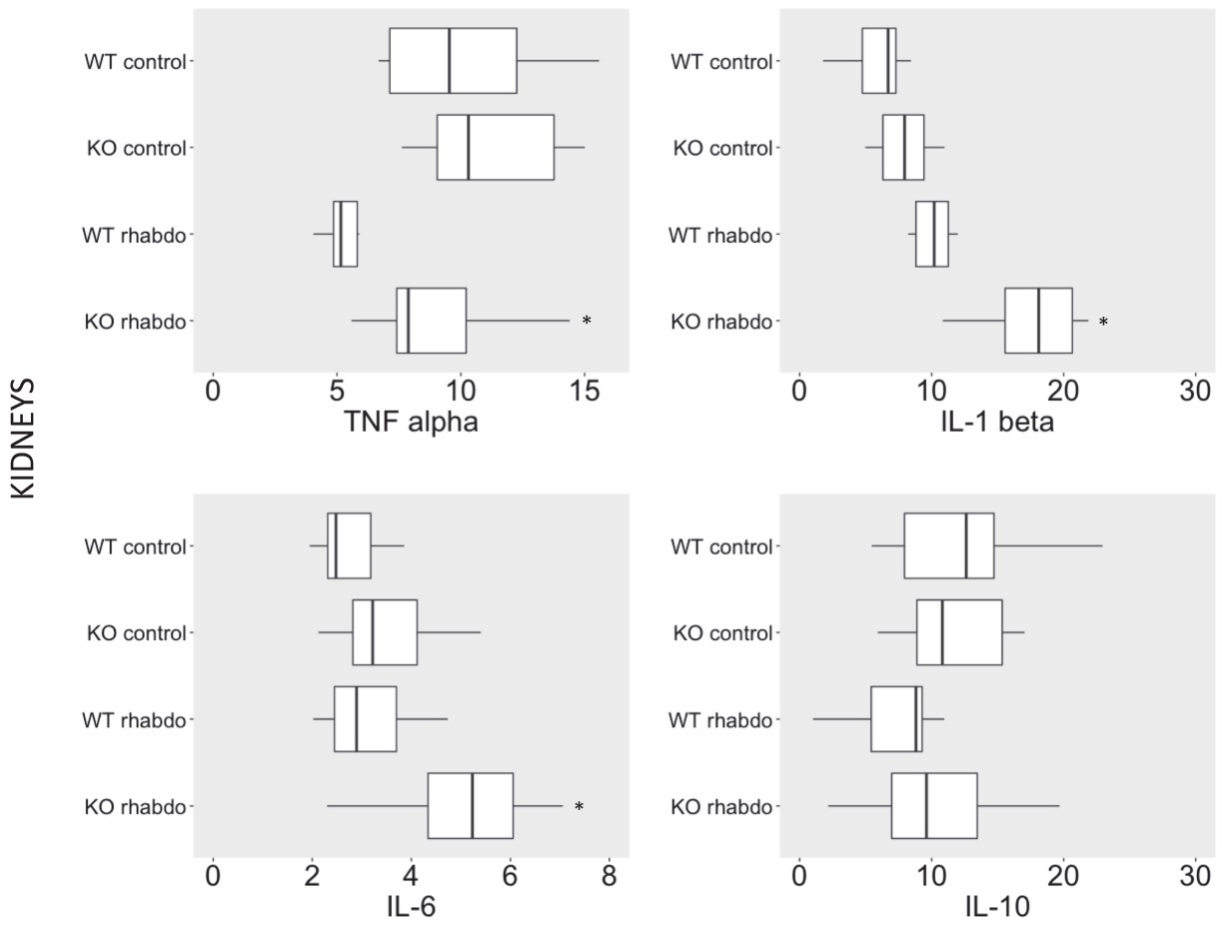
TNFα (pg/ml), IL-1β (pg/ml), IL-6 (pg/ml) and IL-10 (pg/ml) levels in kidney samples from wild-type and CRAMP^-/-^ mice (n = 8-12 animals per group).

TNFα, IL-1β and IL-6 levels were significantly increased in kidney tissue from CRAMP^-/-^ mice subjected to the rhabdomyolysis model compared with the wild-type counterparts (p < 0.01, p = 0.02 and p = 0.03, respectively; **Figure 4**). No differences in IL-10 levels were detected in kidney samples from wild-type and CRAMP^-/-^ mice subjected to the rhabdomyolysis model (**Figure 4**).

### NGAL, β-defensin 1, β-defensin 3 and α-defensin 5 levels in the kidney and distant organs

**Figure 5:**
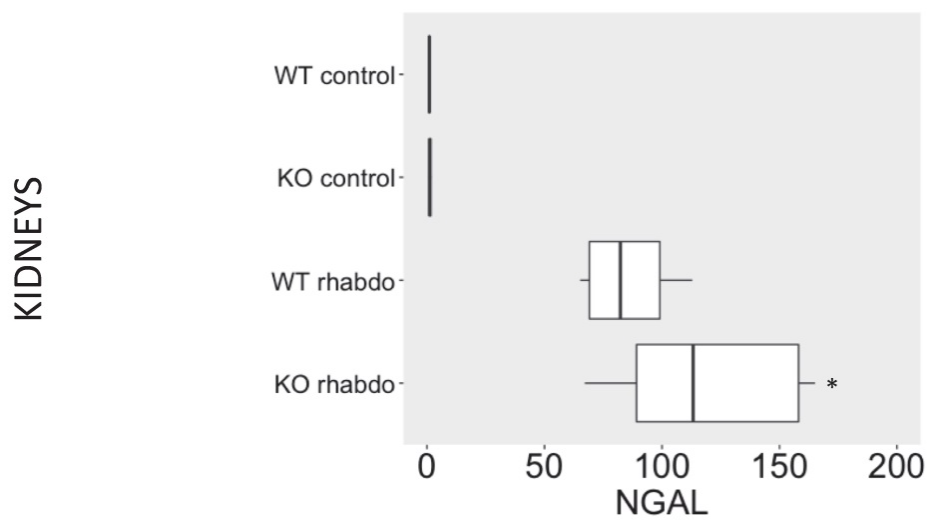
NGAL (ng/ml/mg of total protein) levels in kidney samples (n = 7-12 animals per group).

**Figure 6:**
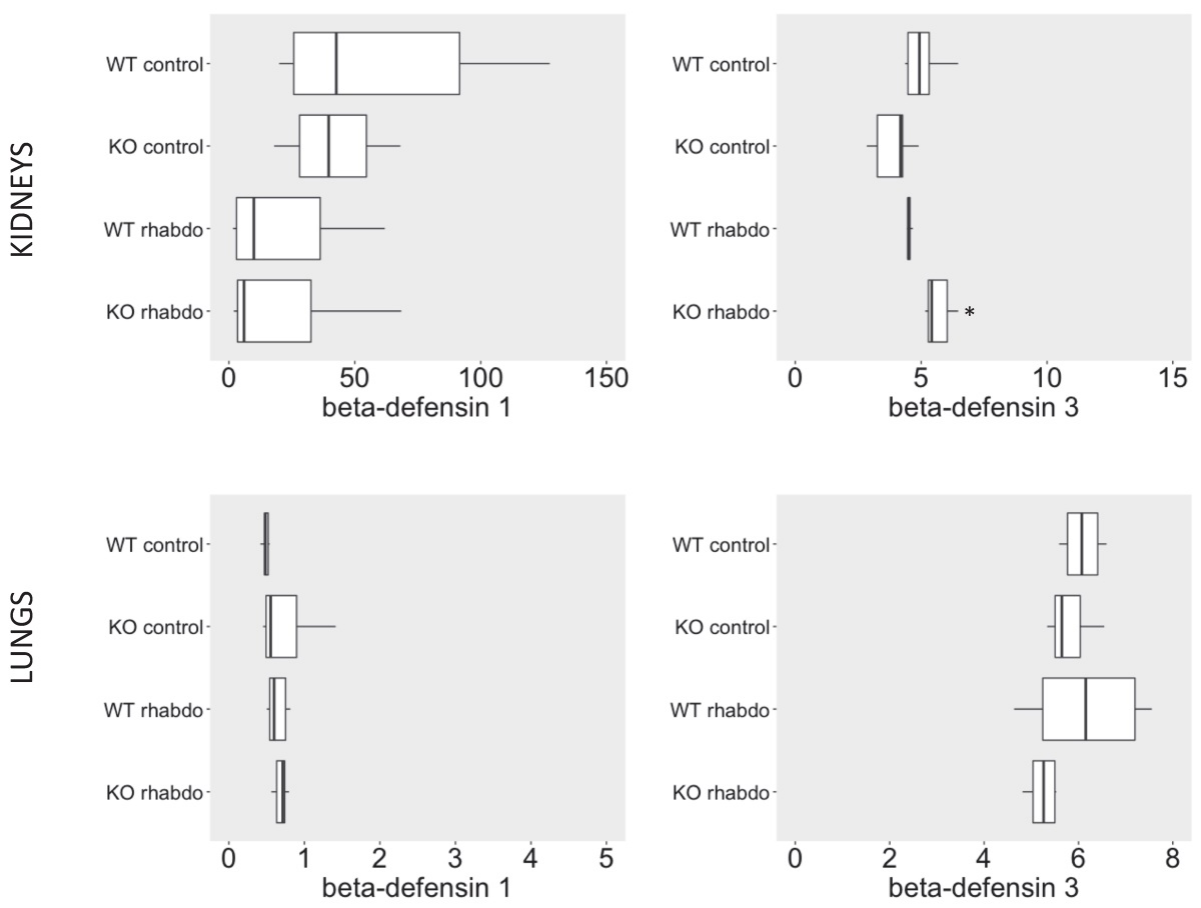
β-defensin 1 (ng/ml/mg of total protein) and β-defensin 3 (ng/L/mg of total protein) levels in kidney samples (above) and lung tissue samples (below) (n = 7-8 animals per group).

**Figure 7:**
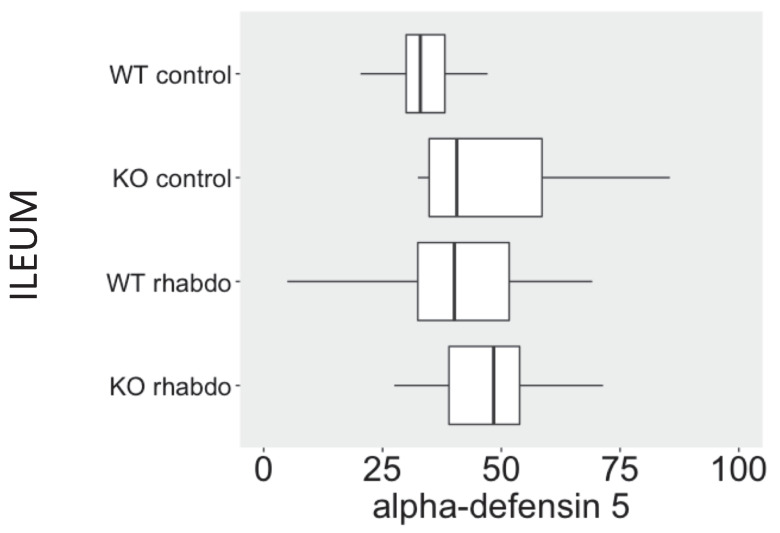
α-Defensin 5 levels (ng/ml/mg of total protein) in ileum samples (n = 8-13 animals per group).

The levels of NGAL in the kidney were higher in the kidney samples from CRAMP -/- submitted to the rhabdomyolysis model, when compared with the wild-type animals in the same conditions (p = 0.031, **Figure 5**). The rhabdomyolysis model did not induce secretion of β-defensin 1. Both in the lungs and in the kidneys, the levels of β-defensin 1 in the rhabdomyolysis groups were similar to the control groups. β-defensin 3 levels, however, were significantly higher in the kidneys of CRAMP -/- mice, when compared with the wild-type controls (p = 0.020) (**Figure 6**). No differences could be found in the levels of α-defensin 5 in the ileum of the study groups (**Figure 7**).

**Figure 8:**
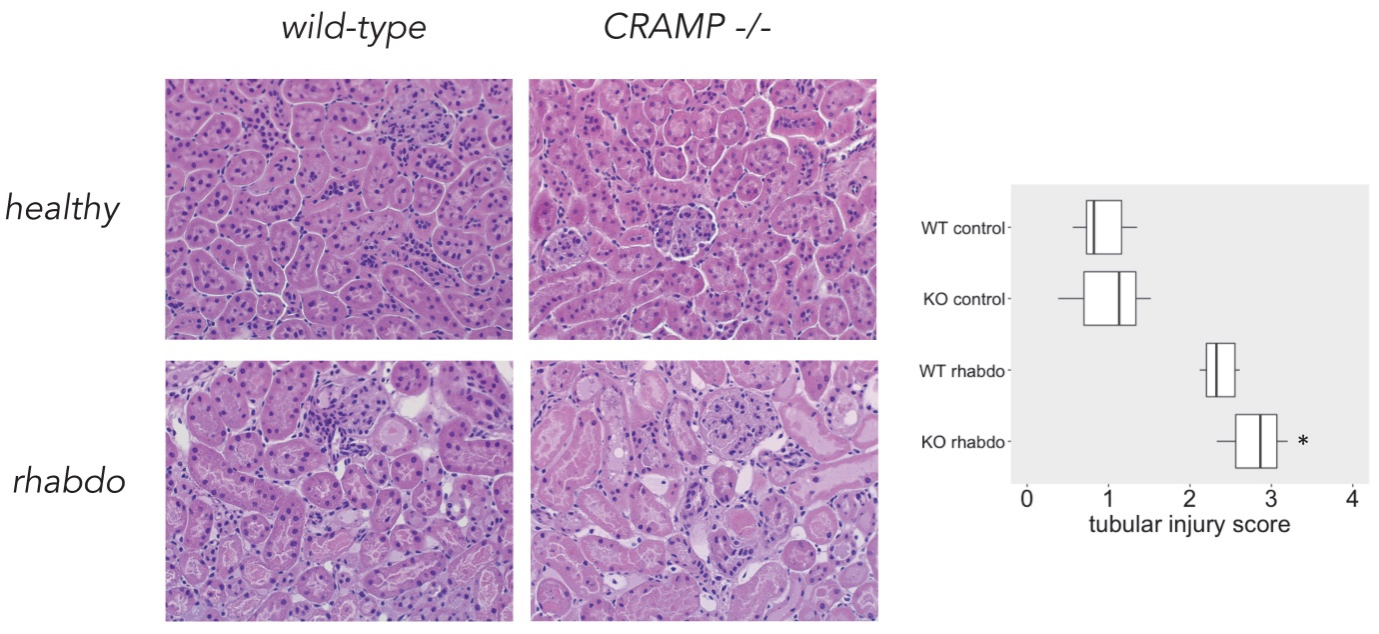
Tubular injury score and microscopical kidney stainings from wild-type and CRAMP^-/-^ animals (n = 8 mice per group).

**Tubular injury score.** Microscopical analysis confirmed that CRAMP^-/-^ mice subjected to the rhabdomyolysis model exhibit more kidney injury than the wild-type counterparts (p = 0.013; **Figure 8**). No difference could be detected among the other study groups.

## Discussion

AKI is defined by a rapid increase in serum creatinine, a decrease in urine output, or both. Serum creatinine, however, is a delayed marker of kidney dysfunction and an insensitive and non-specific marker of injury. In fact, the molecular and cellular responses that accompany sepsis-induced AKI and are secondary to rhabdomyolysis-induced AKI are different, and the use of serum creatinine alone to identify tubular injury is a misleading oversimplification of renal disease due to AKI 21. Although CRAMP^-/-^ mice are more resistant to the cecal ligation and puncture sepsis model, these mice were more sensitive to the rhabdomyolysis model AKI in this study. No differences were detected between the wild-type and CRAMP^-/-^ mice when urea, creatinine, CPK and several electrolytes were measured in the plasma of rhabdomyolysis-induced groups.

NGAL has been investigated as a sensitive and specific biomarker for renal injury 22; however, NGAL levels can be increased in many other inflammatory-associated conditions 23. In contrast to serum creatinine, NGAL does not measure the functional state of the kidney 21. However, compared with serum creatinine, NGAL displays kinetics in the plasma and urine that are more useful as a biomarker for AKI because it is rapidly transcribed following injury and its levels rapidly reverse with relief of the stimulus, exhibiting a dose-dependent response to damage 21. In the current study, we found higher levels of NGAL in the kidney samples of CRAMP -/- animals submitted to the rhabdomyolysis model, when compared to the other study groups. We could not obtain urine samples in our study as most of the animals had developed anuria shortly after induction of AKI.

Interestingly, we found no differences between the levels of TNFα, IL-1β, IL-6 or IL-10 in the plasma of wild-type and CRAMP^-/-^ mice subjected to the rhabdomyolysis model. The only exception was that healthy CRAMP^-/-^ animals exhibited significantly higher TNFα plasma levels than healthy wild-type mice (p < 0.01). In our opinion, this suggests that CRAMP^-/-^ mice might be more susceptible to autoimmune disorders and aging.

This is the first time we have investigated these cytokines in the rhabdomyolysis model; however, in a previously published study of the sepsis model, we found higher levels of IL-1β, IL-6 and MCP-1 in CRAMP^-/-^ septic mice at 8 hours following CLP in comparison with wild-type septic animals 16. Interestingly, when the same cytokines were measured in kidney samples, the TNFα IL-1β and IL-6 levels were significantly higher in CRAMP^-/-^ mice subjected to the rhabdomyolysis model when compared with the wild-type counterparts, putting in evidence that this experimental model induces severe kidney damage without significant systemic inflammatory response.

Among the defensins evaluated as a marker for AKI in this study, β-defensin 1 secretion was not stimulated in the rhabdomyolysis model. By the other hand, β-defensin 3 levels in the kidneys of CRAMP^-/-^ mice subjected to rhabdomyolysis were significantly higher than in wild-type animals similarly induced (p = 0.020).

β-defensin 3, thus, together with NGAL, TNFα, IL-1β and IL-6 are the proinflammatory mediators directly responsible for the worst outcome of CRAMP^-/-^ mice subjected to the rhabdomyolysis model. Histological analysis confirmed that the kidneys of CRAMP -/- subjected to the rhabdomyolysis exhibit more damage, when compared with the wild-type mice.

α-Defensin 5 is produced by Paneth cells in the small bowel. No differences in α-defensin 5 levels were detected between CRAMP^-/-^ and wild-type animals in all the study groups.

The fact that CRAMP^-/-^ mice succumb more rapidly than wild-type mice to the rhabdomyolysis model is probably multifactorial. It is possible that other differences in the cytokine profile exist at an earlier or later time point; however, the systemic inflammatory response in this experimental model is certainly mild. We believe that while in the sepsis model mortality is strongly related to systemic inflammation, in the rhabdomyolysis model the mice die as a consequence of kidney failure. The lungs and the ileum remain protected from inflammatory damage, as indicated by the lack of α- and β-defensin elevation at these sites in the rhabdomyolysis model. There is no multiple organ failure, and the organ damage is localized to and affects only the skeletal muscle and the kidneys.

The intriguing antagonistic cellular responses regulated by CRAMP remain poorly understood; however, cytokines and AMPs clearly form a complex signaling web that plays a major role in health and disease. A deeper comprehension of AMP biology will certainly illuminate the fundamental cellular decisions in these and other complex scenarios.

## References

[1] Agarwal A, Dong Z, Harris R, Murray P, Parikh SM, Rosner MH (2016). Cellular and Molecular Mechanisms of AKI. J Am Soc Nephrol.

[2] Honore PM, Jacobs R, Hendrickx I, Bagshaw SM, Joannes-Boyau O, Boer W (2015). Prevention and treatment of sepsis-induced acute kidney injury: an update. Ann Intensive Care.

[3] Rabb H, Griffin MD, McKay DB, Swaminathan S, Pickkers P, Rosner MH (2016). Inflammation in AKI: Current Understanding, Key Questions, and Knowledge Gaps. J Am Soc Nephrol.

[4] Martensson J, Bellomo R (2016). Pathophysiology of Septic Acute Kidney Injury. Contrib Nephrol.

[5] Peerapornratana S, Manrique-Caballero CL, Gomez H, Kellum JA (2019). Acute kidney injury from sepsis: current concepts, epidemiology, pathophysiology, prevention and treatment. Kidney Int.

[6] Jang HR, Rabb H (2015). Immune cells in experimental acute kidney injury. Nat Rev Nephrol.

[7] Andrade-Oliveira V, Foresto-Neto O, Watanabe IKM, Zatz R, Camara NOS (2019). Inflammation in Renal Diseases: New and Old Players. Front Pharmacol.

[8] Gomez H, Kellum JA (2016). Sepsis-induced acute kidney injury. Curr Opin Crit Care.

[9] Bosch X, Poch E, Grau JM (2009). Rhabdomyolysis and acute kidney injury. N Engl J Med.

[10] Panizo N, Rubio-Navarro A, Amaro-Villalobos JM, Egido J, Moreno JA (2015). Molecular Mechanisms and Novel Therapeutic Approaches to Rhabdomyolysis-Induced Acute Kidney Injury. Kidney Blood Press Res.

[11] Pinheiro da Silva F, Machado MC (2012). Antimicrobial peptides: clinical relevance and therapeutic implications. Peptides.

[12] Becknell B, Schwaderer A, Hains DS, Spencer JD (2015). Amplifying renal immunity: the role of antimicrobial peptides in pyelonephritis. Nat Rev Nephrol.

[13] Chromek M (2015). The role of the antimicrobial peptide cathelicidin in renal diseases. Pediatr Nephrol.

[14] Chromek M, Slamova Z, Bergman P, Kovacs L, Podracka L, Ehren I (2006). The antimicrobial peptide cathelicidin protects the urinary tract against invasive bacterial infection. Nat Med.

[15] Chromek M, Arvidsson I, Karpman D (2012). The antimicrobial peptide cathelicidin protects mice from Escherichia coli O157:H7-mediated disease. PLoS One.

[16] Severino P, Ariga SK, Barbeiro HV, de Lima TM, de Paula Silva E, Barbeiro DF (2017). Cathelicidin-deficient mice exhibit increased survival and upregulation of key inflammatory response genes following cecal ligation and puncture. J Mol Med (Berl).

[17] Heyman SN, Lieberthal W, Rogiers P, Bonventre JV (2002). Animal models of acute tubular necrosis. Curr Opin Crit Care.

[18] Ortiz A, Sanchez-Nino MD, Izquierdo MC, Martin-Cleary C, Garcia-Bermejo L, Moreno JA (2015). Translational value of animal models of kidney failure. Eur J Pharmacol.

[19] Gois PHF, Canale D, Volpini RA, Ferreira D, Veras MM, Andrade-Oliveira V (2016). Allopurinol attenuates rhabdomyolysis-associated acute kidney injury: Renal and muscular protection. Free Radic Biol Med.

[20] Ferreira D, de Braganca AC, Volpini RA, Shimizu MHM, Gois PHF, Girardi ACC (2019). Vitamin D deficiency is a potential risk factor for lipid Amphotericin B nephrotoxicity. PLoS Negl Trop Dis.

[21] Desanti De Oliveira B, Xu K, Shen TH, Callahan M, Kiryluk K, D'Agati VD (2019). Molecular nephrology: types of acute tubular injury. Nat Rev Nephrol.

[22] Antonucci E, Lippi G, Ticinesi A, Pigna F, Guida L, Morelli I (2014). Neutrophil gelatinase-associated lipocalin (NGAL): a promising biomarker for the early diagnosis of acute kidney injury (AKI). Acta Biomed.

[23] Umbro I, Gentile G, Tinti F, Muiesan P, Mitterhofer AP (2016). Recent advances in pathophysiology and biomarkers of sepsis-induced acute kidney injury. J Infect.

